# Structural Insights into the Recovery of Aldolase Activity in *N*-Acetylneuraminic Acid Lyase by Replacement of the Catalytically Active Lysine with γ-Thialysine by Using a Chemical Mutagenesis Strategy

**DOI:** 10.1002/cbic.201200714

**Published:** 2013-02-18

**Authors:** Nicole Timms, Claire L Windle, Anna Polyakova, James R Ault, Chi H Trinh, Arwen R Pearson, Adam Nelson, Alan Berry

**Affiliations:** a Astbury Centre for Structural Molecular Biology, University of Leeds, Garstang BuildingLeeds, LS2 9JT (UK); b School of Molecular and Cellular Biology, University of Leeds, Garstang BuildingLeeds, LS2 9JT (UK); c School of Chemistry, University of LeedsLeeds, LS2 9JT (UK)

**Keywords:** aldolases, enzyme catalysis, N-acetylneuraminic acid lyase, thialysine, unnatural amino acids

## Abstract

Chemical modification has been used to introduce the unnatural amino acid γ-thialysine in place of the catalytically important Lys165 in the enzyme *N*-acetylneuraminic acid lyase (NAL). The *Staphylococcus aureus nan*A gene, encoding NAL, was cloned and expressed in *E. coli*. The protein, purified in high yield, has all the properties expected of a class I NAL. The *S. aureus* NAL which contains no natural cysteine residues was subjected to site-directed mutagenesis to introduce a cysteine in place of Lys165 in the enzyme active site. Subsequently chemical mutagenesis completely converted the cysteine into γ-thialysine through dehydroalanine (Dha) as demonstrated by ESI-MS. Initial kinetic characterisation showed that the protein containing γ-thialysine regained 17 % of the wild-type activity. To understand the reason for this lower activity, we solved X-ray crystal structures of the wild-type *S. aureus* NAL, both in the absence of, and in complex with, pyruvate. We also report the structures of the K165C variant, and the K165-γ-thialysine enzyme in the presence, or absence, of pyruvate. These structures reveal that γ-thialysine in NAL is an excellent structural mimic of lysine. Measurement of the pH-activity profile of the thialysine modified enzyme revealed that its pH optimum is shifted from 7.4 to 6.8. At its optimum pH, the thialysine-containing enzyme showed almost 30 % of the activity of the wild-type enzyme at its pH optimum. The lowered activity and altered pH profile of the unnatural amino acid-containing enzyme can be rationalised by imbalances of the ionisation states of residues within the active site when the p*K*_a_ of the residue at position 165 is perturbed by replacement with γ-thialysine. The results reveal the utility of chemical mutagenesis for the modification of enzyme active sites and the exquisite sensitivity of catalysis to the local structural and electrostatic environment in NAL.

## Introduction

Enzymes are typically built of the 20 proteogenic amino acids and are phenomenally powerful catalysts that may increase reaction rates over 10^18^-fold.^1^, ^2^ Their catalytic function stems from the range of side-chain chemistries available which can be supplemented by the ability to bind and recruit metal ions or other cofactors. However, in some cases, nature has expanded the catalytic repertoire of enzymes by using noncanonical amino acids incorporated into the polypeptide chain, either by genetic encoding (such as selenocysteine[3] and pyrrolysine[4]) or by post-translational modifications. Examples of catalytically important residues incorporated by post-translational modifications include the quinone cofactors such as 2,4,5-trihydroxyphenylalanine quinone (TPQ) in amine oxidase, lysyl tyrosylquinone (LTQ) in lysyl oxidase and tryptophan tryptophylquinone (TTQ) in methylamine dehydrogenase,^5^, ^6^ formylglycine in the type I sulfatases,[7] 4-methylidene imidazole-5-one (MIO) in aminomutases[8] and the N-terminal pyruvoylphenylalanine in yeast histidine decarboxylase.[6] Protein engineers have exploited rational site-directed mutagenesis and directed evolution to alter many properties of enzymes including their specificity, stability and stereochemistry.^9^, ^10^ More recently computer-aided enzyme design, coupled with directed evolution has made significant advances in opening the way to new designer enzymes.^11^–^13^ However these approaches have remained limited to the use of the 20 proteogenic amino acids.

**Scheme 1:**
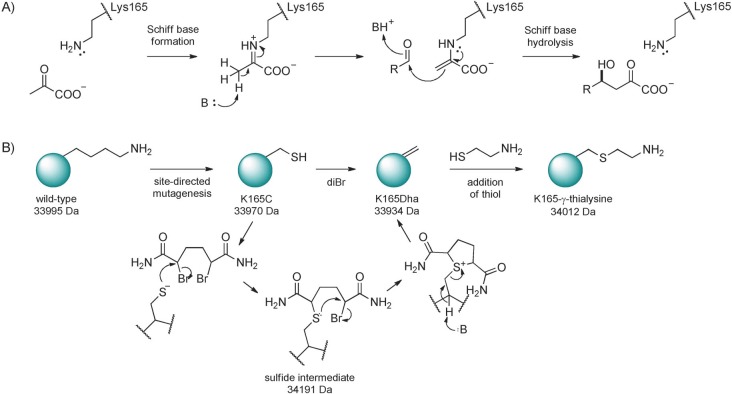
Mechanism and chemical modification of *N*-acetylneuraminic acid lyase. A) The enzymatic reaction mechanism showing the involvement of Lys165 in forming the pyruvate Schiff base. B) The scheme for the chemical modification[16] to introduce unnatural amino acids through dehydroalanine illustrated by the conversion of Lys165 to γ-thialysine. Molecular masses shown illustrate the expected masses for modification of the *S. aureus* NAL.

Two methods have recently been developed that enable the synthetic biologist to use unnatural amino acids to construct novel enzyme active sites: one using the ribosome in conjunction with new, orthogonal tRNA/tRNA synthetase (tRNAS) pairs^14^, ^15^ and the other, using chemical modification, to introduce new amino acid side chains.[16] The use of orthogonal tRNA/tRNAS pairs has found growing use,[17] but the yields of recombinant protein containing the unnatural amino acid can be disappointing. In contrast, expression of the natural polypeptide chain may yield high levels of protein, but the low efficiency[18] and lack of specificity[19] of many chemical modification strategies limits their usefulness. In 1966, Koshland noted the use of dehydroalanine (Dha) in “chemical mutagenesis”[20] and recently Chalker and Davis have devised an efficient double alkylation-elimination method to generate Dha in an enzyme and convert it to an unnatural amino acid (Scheme 1).[16]

Here we use this method[16] to convert the catalytically essential lysine of the enzyme *N*-acetylneuraminic acid lyase (NAL) to generate a nonproteogenic amino acid and we analyse the resulting enzyme to explain the structural origins and basis of its catalytic activity.

NAL catalyses the reversible aldol condensation of *N*-acetylmannosamine with pyruvate to yield the sialic acid, *N*-acetylneuraminic acid (Neu5Ac). We have previously shown the *E. coli* enzyme to be an attractive target for rational protein engineering and directed evolution.^21^–^23^ NAL is a class I aldolase, functioning through the formation of an enzyme–pyruvate Schiff base formed with Lys165 which carries out a nucleophilic attack on the aldehyde carbon of the open-chain form of the *N*-acetylmannosamine (Scheme 1).[24] Here, using the NAL from *Staphylococcus aureus*, we demonstrate quantitative conversion of a cysteine residue, site-specifically into the nonproteogenic amino acid γ-thialysine, chosen as a mimic of the natural catalytic lysine residue at position 165, using the double alkylation-elimination method[16] and that high yields of an active, modified NAL can be purified. We then characterise the resulting enzyme activity, analyse the pH-dependency of catalysis and use X-ray crystallography to investigate the structural basis of the changes brought about by modifying the enzyme. These results open the way to the construction of novel aldolase active sites containing unnatural amino acids for new chemistries.

## Results and Discussion

The chemical insertion of a nonproteogenic amino acid into the active site of NAL requires the conversion of a cysteine residue into dehydroalanine followed by conjugate addition of a thiol to generate the new amino acid side chain (Scheme 1).[16] Initial work on the *E. coli* NAL revealed that removal of the four natural cysteine residues in the enzyme (Cys82, Cys118, Cys238 and Cys270) by mutagenesis to either serine or alanine residues produced enzymes with kinetic properties identical with the wild-type enzyme (see Table S1 in the Supporting Information). Subsequent mutation of Lys165 to a cysteine residue, produced an enzyme with only low activity, as expected, but this mutant enzyme was unsuitable for chemical modification because of its poor long-term solubility, slowly precipitating over a period of 1–2 days. We therefore scanned the protein sequence databases for natural, cysteine-free, NAL enzymes. A putative sequence from *S. aureus* was identified (Uniprot accession number Q2G160) and we amplified the gene (*nan*A) by PCR (Table S2) and cloned it into the expression vector pKK223-3. After induction of protein expression with IPTG, a band of 34 kDa was seen on SDS-PAGE corresponding to the size of protein expected from the DNA sequence. The protein was purified using a His_6_ tag appended to the N terminus of the protein, in the same manner as used previously for the *E. coli* enzyme.[22] The purified protein was shown to have NAL activity and the kinetic parameters determined were similar to those of the *E. coli* NAL (Table 1). Positive mode ESI-MS of the enzyme identified its molecular mass as 33 992±0.48 Da, in agreement with the expected mass calculated from the amino acid sequence (33 995 Da).

**Table 1:** Steady-state kinetic parameters of wild-type and variant *S. aureus* NAL. Steady-state kinetic parameters for the cleavage of *N*-acetylneuraminic acid (Neu5Ac) by wild-type and variant *S. aureus* NAL determined using an LDH-coupled enzyme assay.^2^,^38^ Data were fitted to the Michaelis–Menten equation and the fitted values±standard error of the fit are shown.

Enzyme	pH	*K*_m_ (Neu5Ac)	*k*_cat_	*k*_cat_/*K*_m_	% of pH 7.4
(assay pH)	[mm]	[min^−1^]	[min^−1^ mm^−1^]	wild-type *k*_cat_/*K*_m_	
wild type	7.4	2.2±0.1	250±5	114	100
K165C	7.4	0.8±0.1	0.08±0.004	0.1	0.09
K165-γ-thialysine pre-gel-filtration	7.4	0.7±0.05	8±0.1	11	10
gel-filtered K165-γ-thialysine	7.4	1.4±0.2	26±0.9	19	17
wild type	6.8	2.4±0.2	260±6	108	95
gel-filtered K165-γ-thialysine	6.8	0.9±0.09	29±0.8	32	28

A mutant *nan*A gene encoding the K165C *S. aureus* NAL was created using site-directed mutagenesis and DNA sequencing confirmed the presence of the mutation. The variant protein was expressed and purified in the same manner as the wild type. The steady-state parameters for Neu5Ac cleavage showed that the K165C variant was severely impaired in catalysis (*k*_cat_/*K*_m_ reduced 720-fold compared with wild-type; Table 1) as would be expected for the removal of the catalytically important Schiff-base-forming residue, and in line with similar studies on other class I aldolases.#x005B;18]

### Chemical modification of K165C to yield γ-thialysine

**Figure 1:**
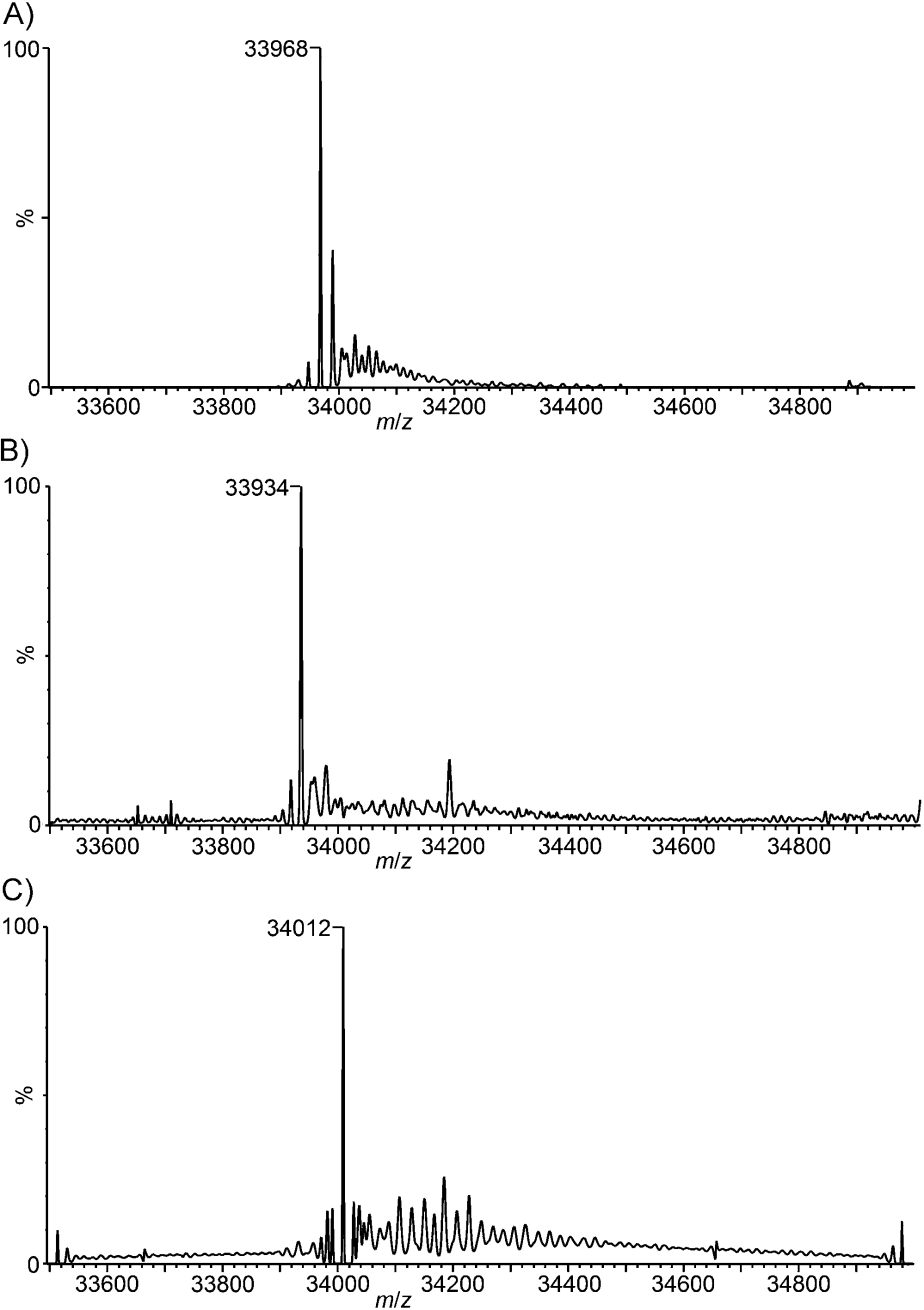
Deconvoluted ESI-mass spectra of A) the K165C variant of *S. aureus* NAL, B) the K165C variant after 1.5 h treatment with 2,5-dibromo-1,6-hexadiamide, the mass found (33 934±0.63 Da) corresponds exactly with that expected (33 934 Da) for the K165Dha enzyme, and C) the K165Dha enzyme after reaction with 2-aminoethanethiol to generate the K165-γ-thialysine enzyme. The molecular mass found (34 012±0.16 Da) corresponds exactly with that expected (34 012 Da) for the chemically modified enzyme. (See Figure S3 for *m/z* spectra).

The purified K165C NAL was chemically modified to introduce the new side chain at position 165. Experiments demonstrated that the cysteine side chain in the native protein was inaccessible to the reagent 2,5-dibromo-1,6-hexadiamide used to generate the dehydroalanine residue. The protein was thus unfolded in 6 m urea prior to treatment with 2,5-dibromo-1,6-hexadiamide for 1.5 h at 37 °C. Under these conditions a double alkylation reaction followed by elimination takes places to generate K165Dha (Scheme 1 B). An ESI mass spectrum taken after 1.5 h revealed a single protein species of molecular mass 33 934±0.63 Da, exactly as expected for the K165Dha variant (33 934 Da, Figure 1), demonstrating the quantitative conversion of Cys165 to dehydroalanine (Dha165).

**Figure 2:**
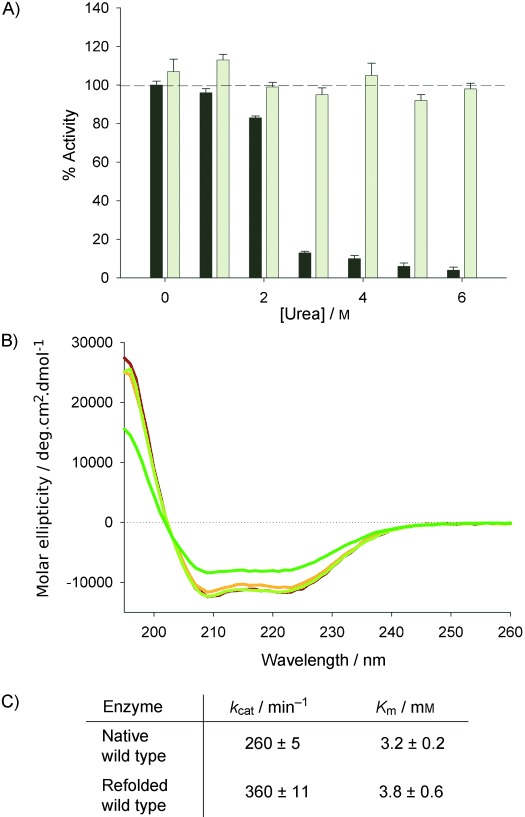
Refolding of *S. aureus* NAL after denaturation in urea. A) *S. aureus* NAL (2 mg mL^−1^) was treated with various concentrations of urea in Tris⋅HCl buffer (50 mm, pH 7.4) and the activity was measured (dark bars). The urea in the sample was then rapidly diluted tenfold with the same buffer and activity was measured again after 10 min (light grey bars). 98±3 % of the wild-type activity is recovered after tenfold dilution from 6 m urea. Similar results were found if rapid dilution is replaced by dialysis in the same buffer (data not shown). B) Far-UV CD spectra of wild-type *S. aureus* NAL (red); refolded wild-type NAL (orange); thialysine-165-containing NAL before gel filtration (dark green) and gel-filtered thialysine-165 NAL (light green). C) The kinetic parameters determined at pH 7.4 for wild-type *S. aureus* NAL before and after refolding.

The dehydroalanine side chain was next converted into γ-thialysine by conjugate addition of 2-aminoethanethiol onto the K165Dha protein. Analysis by ESI-MS after two hours incubation of the enzyme with 2-aminoethanethiol revealed quantitative conversion of K165Dha into a single peak corresponding to a protein of 34 012±0.16 Da, in excellent agreement with that expected for the K165-γ-thialysine enzyme (expected mass 34 012 Da). The incorporation of the modification at position 165 was also confirmed by peptide sequencing using LC-MS/MS after tryptic digestion of the protein (see Figure S4). The K165-γ-thialysine enzyme was then refolded by dialysis under conditions shown to regenerate 100 % of native activity for refolding of the denatured wild-type enzyme (Figure 2 A and C). Following refolding, the K165-γ-thialysine enzyme regained significant activity compared with the K165C variant (Table 1) Using this method up to 50 mg of enzyme was successfully modified.

The regain of approximately 10 % of the wild-type activity (based on *k*_cat_/*K*_m_ values) upon replacement of a catalytic lysine residue with γ-thialysine is in line with reports of similar replacements carried out previously by direct alkylation of an introduced cysteine at the site of catalytic lysine residues.^18^, ^25^–^28^ However in a number of those cases the low activity recovered was explained by incomplete conversion. Here, the lower *k*_cat_ values for the K165-γ-thialysine-containing enzyme cannot be explained by incomplete conversion of K165Dha since there is no evidence of K165Dha or unmodified enzyme in the ESI-mass spectrum. We therefore sought further evidence to evaluate whether structural differences between NAL containing lysine or γ-thialysine at position 165 might be responsible for the lower activity of the chemically modified enzyme. As a prelude to X-ray crystallographic studies we further purified the chemically modified γ-thialysine-containing NAL by gel filtration. Unlike the wild-type *S. aureus* NAL, the chemically modified protein eluted in two peaks: one active fraction at the elution volume expected for a protein of approximately 34 kDa; and another at the void volume of the column, due to aggregated protein. Removal of this material resulted in an approximate doubling of the specific activity of the modified enzyme, to 17 % (based on *k*_cat_/*K*_m_ values) of the activity of the wild-type enzyme (Table 1) and far-UV CD spectroscopy suggests that the modified enzyme was correctly folded as its spectrum is identical to that of the wild-type enzyme (Figure 2 B).

### Crystallographic studies

The wild-type *S. aureus* NAL was crystallised and the structure solved using molecular replacement methods (PDB ID: 4AHP). In addition, the structure of the enzyme–pyruvate complex (PDB ID: 4AH7) was also determined. Finally, we solved the structure of the K165C variant (PDB ID: 4AHQ), and the K165-γ-thialysine enzyme with (PDB ID: 4AMA), and without (PDB ID: 4AHO) bound pyruvate. All structures were solved in space group *P*2_1_2_1_2_1_ (see Table S5 for structural data).

**Figure 3:**
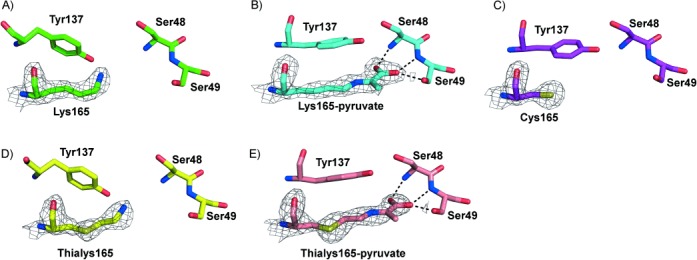
X-ray crystal structures of active-site residues of wild-type and chemically modified *S. aureus* NAL. A) Wild-type apoenzyme (4AHP). B) The Schiff base of Lys165 with pyruvate (4AH7). The pyruvate carboxylate group interacts with the backbone amides of Ser48 and Ser49 and the side-chain hydroxyl group of Ser49, identical to the interactions with Ser47 and Thr48 previously seen in the pyruvate complex of the *E. coli* enzyme.[29] C) The structure of the active site of the K165C enzyme (4AHQ). D) The active site of the γ-thialysine-165 enzyme (4AHO). E) The structure of the Schiff base formed between pyruvate and γ-thialysine at position 165 in the chemically modified enzyme (4AMA). The carboxylate makes the same interactions as for the wild-type *S. aureus* enzyme. In all cases the structure of subunit A of the tetramer is illustrated and electron density around residue 165 is mapped at the 1.4 *σ* level. In each case the atoms are coloured by atom type with the carbon atoms coloured green in the wild-type structure, cyan in the wild-type pyruvate complex, purple in the K165C structure, yellow in the thialysine-containing NAL and salmon in the thialysine-NAL–pyruvate complex.

The crystal structure of the *S. aureus* NAL shows that the enzyme adopts a TIM-(β/α)_8_-barrel tertiary structure identical to that previously observed for the *E. coli* (PDB ID: 2WO5)[29] and *Haemophilus influenzae* (PDB ID: 1F5Z)[30] NALs, with RMSD values of 1.36 and 0.84 Å for alignments of the subunit alpha-carbons of the *S. aureus* structure with the *E. coli* and *H. influenzae* enzymes, respectively. Comparison of the *E. coli* (PDB ID: 2WNN)[29] and *S. aureus* (4AH7) enzymes in complex with pyruvate showed that the pyruvate is covalently linked to the enzyme as a Schiff base and makes identical interactions with the backbone amides of Ser48 and Ser49 and also with the side-chain hydroxyl group of Ser49 (equivalent to Ser47 and Thr48 in the *E. coli* enzyme; Figure 3 A and B). The effect of Schiff base formation in the *S. aureus* enzyme is also similar to that found with the *E. coli* enzyme, namely that it causes ordering of the loop region (138–146) which is not resolved in apo structures. This results in a repositioning of Tyr137, a residue noted as being involved in substrate stabilisation and/or catalysis.[31]

The structure of the K165C variant showed no significant changes in the overall structure of the protein compared with the wild-type enzyme (RMSD over all Cα atoms=0.34 Å). However, the observed electron density for residue 165 did not extend far enough for a lysine residue and was better fitted by a cysteine residue (Figure 3 C) as expected. Interestingly, the K165C structure more closely resembled the enzyme–pyruvate complexes than the apo structures since the loop region (138–146), not generally resolved in apo structures, could be clearly seen in the structure of the K165C variant and the side chain of Tyr137 was also positioned more similarly to that in the pyruvate complexes (Figure 3 C). Calculation of side-chain solvent accessibility for the K165C variant showed that the cysteine is inaccessible explaining the need to unfold the protein prior to chemical modification. Despite soaking K165C crystals with up to 100 mm pyruvate, we were never able to observe electron density for substrate in the enzyme active site.

The 3D structure of the K165-γ-thialysine enzyme was solved at 2 Å resolution. Initial modelling of Lys165 in all four subunits revealed a strong positive electron density around the gamma carbon of the side chain of residue 165, thus indicating the presence of a more electron-dense atom. Subsequent replacement with γ-thialysine side chains correctly accounted for this density in the *F*_obs_−*F*_calcd_ map and showed the presence of γ-thialysine in all four subunits of the enzyme (Figure 3 D). The chemical modification of the enzyme has not altered its overall fold. Alignment of all of the Cα atoms of the K165-γ-thialysine structure with the wild-type structure resulted in an RMSD value of only 0.13 Å. Interestingly, the crystal structure of modified enzyme showed no evidence of d-thialysine at position 165. This is despite the fact that the thialysine side chain is generated by Michael addition onto the planar dehydroalanine which, in model peptides[32] at least, can result in thiol attack onto either face of the double bond producing a mixture of d- and l-modified amino acids. Since burial of residue 165 in the *S. aureus* NAL active site necessitated unfolding of the enzyme in 6 m urea in order that modification could take place, it is likely that a mixture of d- and l-thialysine-modified NAL will be generated, although residual structure in the unfolded state and the chiral nature of the polypeptide backbone might impose some steric constraints on the modification reaction. We hypothesise therefore that both stereoisomers are formed but that the d-thialysine-modified protein does not correctly refold, and is removed by the gel-filtration step.

The positioning of the nitrogen atom in lysine/γ-thialysine-165 may be crucially important for the catalytic ability of the enzyme. The γ-thialysine residue is found to be in a fully extended *anti* configuration within the active site, as is observed with Lys165 in the wild-type enzyme. However the presence of the sulfur atom in the γ-thialysine side chain alters both the bond lengths around the gamma atom of the residue and the bond angles of the side chain. In the wild-type enzyme the average Cβ–Cγ and Cγ–Cδ bond lengths in Lys165 in the four polypeptide chains in the tetramer are both 1.5 Å and the Cβ–Cγ–Cδ bond angle is 110.1°, in excellent agreement with the values of 1.53 Å, 1.54 Å and 110.0° found in free lysine.[33] The average Cβ–Sγ and Sγ–Cδ bond lengths in the four γ-thialysines in the tetramer are 1.8 Å and the Cβ–Sγ–Cδ bond angle is 97.2° (compared with 1.82 Å, 1.81 Å and 102.9° in free γ-thialysine[34]). These changes result in the overall “length” of the residue at position 165 (as measured by the Cα-NZ distance) increasing from 6.1 Å for Lys165 to 6.4 Å for γ-thialysine-165. Therefore the amino group of the residue at position 165 is moved only approximately 0.3 Å towards the central axis of the (β/α)_8_-barrel of the enzyme, due to the increased bond lengths in thialysine, counteracted by the tighter Cβ–Sγ–Cδ bond angle.

Soaking the crystals of K165-γ-thialysine NAL with 100 mm pyruvate allowed the structure of the pyruvate complex of this enzyme to be solved (Figure 3 E). There was clear continuous electron density at the 1.5 *σ* level linking the residue at position 165 with the pyruvate, revealing that the unnatural γ-thialysine side chain is able to form a Schiff base with the pyruvate as part of the catalytic cycle. In this complex the pyruvate makes identical contacts with the enzyme as in the wild-type–pyruvate complexes; Tyr137 adopts the substrate-bound conformation, and the loop 138–146 becomes ordered (Figure 3 E).

**Figure 4:**
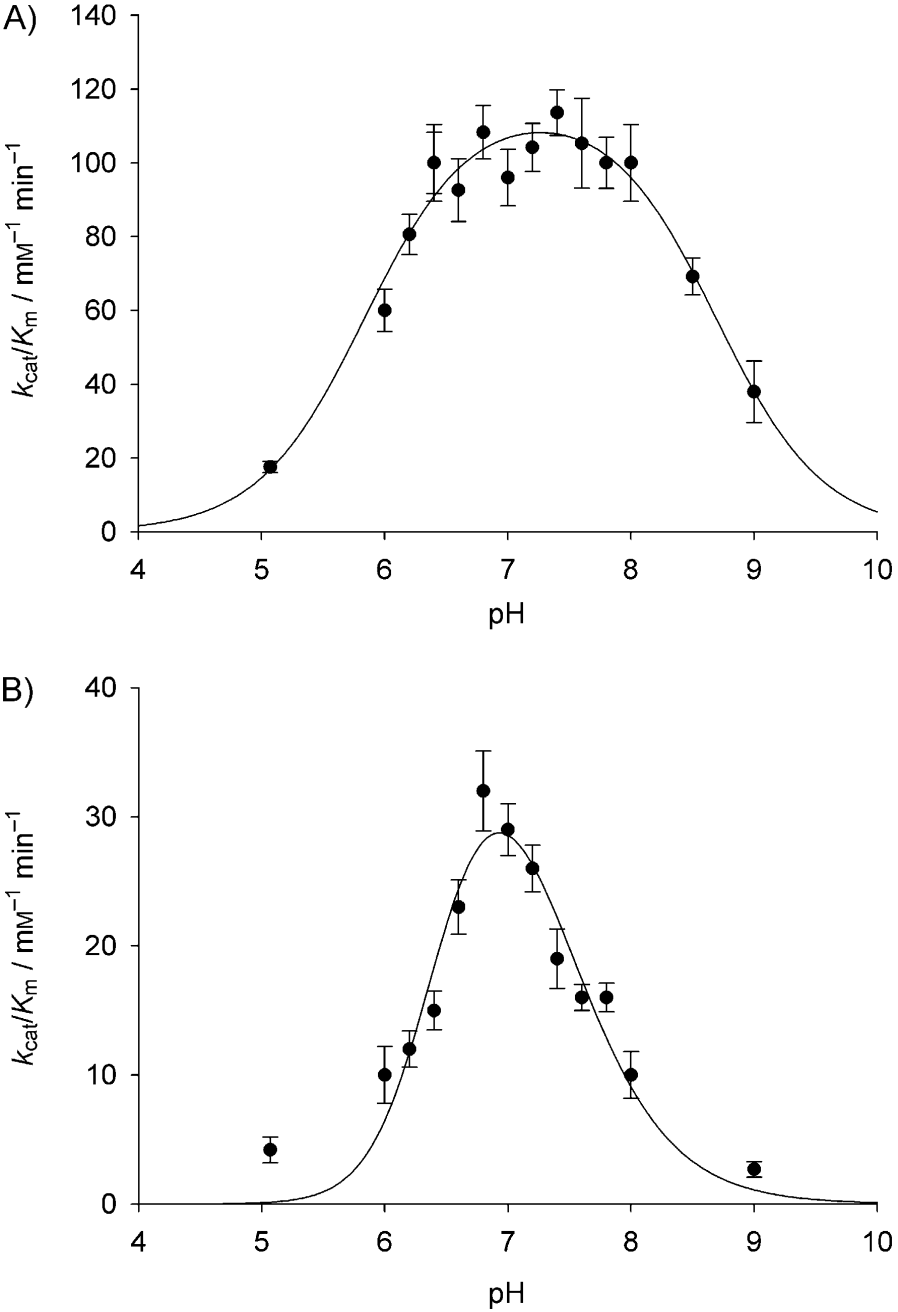
pH profiles of *k*_cat_/*K*_m_ values for NAL activity of A) wild-type *S. aureus* NAL, and B) 165-γ-thialysine NAL. Kinetic parameters were measured for the enzymes using the standard NAL assay^22^, ^38^ at 30 °C in a three component buffer system.[39] The data are fitted to bell-shaped pH profiles.

In all these aspects this modified enzyme behaves as the wild-type enzyme and the active-site geometry is virtually identical with that of the wild-type–pyruvate complex. Gross geometric constraints or structural alterations therefore cannot account for the difference in enzyme activity between the γ-thialysine-165 enzyme and the wild type. As well as the slight structural alterations imposed by the chemical modification of the side chain, the inclusion of a sulfur atom in γ-thialysine affects the p*K*_a_ of the amino group of the residue, lowering its p*K*_a_ by about 1.15 pH units when compared with lysine.[35] To investigate how changing the pH of the enzyme assay affects the activity of the thialysine-165 variant, we measured the pH-activity profile of both wild-type and thialysine-containing *S. aureus* NAL. The pH profile for the wild-type *S. aureus* enzyme (Figure 4 A and Figures S6 and S7) shows a typical bell-shaped profile with a pH optimum of 7.4 and two p*K*_a_ values of 5.8 and 8.7. Since these apparent p*K*_a_ values are almost 3.5 pH units apart the theoretical maximum *k*_cat_/*K*_m_ value (obtained from the fit of the data) of 116 min^−1^ mm^−1^ aligns well with the *k*_cat_/*K*_m_ value measured (at pH 7.4) for the wild-type enzyme of 114 min^−1^ mm^−1^ (Table 1). It has not been possible to identify residues responsible for these p*K*_a_ values. The active site of the enzyme contains a predominance of tyrosine residues among those side-chains which are ionisable but despite the fact that many of these residues are predicted by PROPKA[36] to have p*K*_a_ values significantly shifted from that of free tyrosine, none correspond exactly to the experimentally determined values.

The pH profile for the γ-thialysine-165 NAL is significantly different to that of the wild-type enzyme (Figure 4 B). The profile is narrower and has a pH optimum at approximately pH 6.8, lower than the value of 7.4 found for the wild-type enzyme. Fitting the data to the standard bell-shaped pH profile reveals two apparent p*K*_a_ values, of 6.84 and 7.12, and a theoretical maximum value of *k*_cat_/*K*_m_ of 71 min^−1^ mm^−1^. In cases where the two p*K*_a_ values are less than 3.5 pH units apart, the theoretical maximum *k*_cat_/*K*_m_ value is never observed since the enzyme is never fully in the correct ionisation state. At its pH optimum of 6.8, the observed *k*_cat_/*K*_m_ value of thialysine-containing NAL is 32 min^−1^ mm^−1^ (Table 1), demonstrating a recovery of approximately 30 % of the wild-type activity. Analysis of the theoretical titration curves for an enzyme with two active site residues with p*K*_a_ values of 6.84 and 7.12 shows that only about 33 % of the enzyme would be in the active form at the pH optimum (see Figure S7). This suggests that the thialysine-containing active site is perfectly primed for catalysis, but that the microenvironment around the active site has been perturbed by the introduction of the thialysine side chain such that the p*K*_a_ values of various active site residues are unbalanced and the full power of catalysis cannot be realised.

Overall, the work described demonstrates the powerful utility of using chemical mutagenesis to derive enzymes with unnatural catalytic side chains. Furthermore, it demonstrates that active enzymes can be easily produced and assayed in such a way that screening libraries of NAL with unnatural amino acids at a variety of locations within the active site is a feasible proposition. From a practical perspective the chemical mutagenesis strategy requires an enzyme with a single cysteine residue and, ideally, one that is solvent exposed for ease of modification. However we have demonstrated that denaturation, followed by chemical modification and refolding is a viable means to generate large quantities of active, unnatural amino acid bearing variants of NAL in sufficient yields for detailed kinetic and structural studies. For the NAL enzyme described here, crystallography reveals alignment of the new catalytic thialysine in the enzyme active site placed in a conformation set up for efficient catalysis. However, the thialysine-containing enzyme does not recover full activity, because the introduction of the sulfur atom in the new residue unbalances the ionisation environment of the active site such that only 33 % of the enzyme is in the correct state for catalysis to occur. Nevertheless, replacement of the natural lysine side chain with a thialysine residue is still consistent with activity and the rate of the reaction is enhanced 360-fold when compared with the inactive K165C variant.

The procedure developed enables the facile production of new NAL enzymes that can contain a repertoire of novel amino acid side chains at position 165 that is almost limitless. Compared with the limitations of mutagenesis, chemical strategies involving dehydroalanine modification are now able to reach their full potential as predicted by Koshland more than 45 years ago. This in turn unlocks the potential, outlined by Brustad and Arnold[37] for the protein engineer to combine the use of unnatural amino acids with the power of directed evolution to expand the realm of catalysis and improve protein fitness for functionality to areas not available to nature.

## Experimental Section

**Cloning and mutagenesis:** The *nan*A gene was amplified from *S. aureus* NCTC 8325 genomic DNA (0.1 ng μL^−1^) by PCR with *Pfu* polymerase using primers designed to incorporate a His_6_ tag at the N terminus of the protein and including EcoRI and PstI restriction sites to allow subcloning (see Table S2 for primer sequences). The PCR product of around 900 bp was gel purified, restricted with EcoRI and PstI and ligated into pKK223-3 (Pharmacia) cut with the same enzymes. The resulting expression vector was designated pKSAnanA. Site-directed mutagenesis was carried out using QuikChange Lightning or QuikChange multisite-directed mutagenesis kits (Stratagene, UK) according to the manufacturer–s guidelines. Primers were designed by using the recommended QuikChange Primer Design Program (Stratagene, UK).

**Expression and purification of NAL:** Wild-type and variant *E. coli* NAL were expressed from the plasmid pK*nan*A-His_6_ as previously described.[22] *S. aureus* NAL (wild-type and variants) was expressed from *E. coli* BL21 (DE3) cells transformed with the plasmid pKSAnanA harbouring the wild-type or mutant *S. aureus* NAL gene, by using the method previously described.[22] Cells were grown in 2YT medium (5 mL) containing ampicillin (50 μg mL^−1^) and inoculated into 2YT medium (2 L) containing ampicillin (50 μg mL^−1^) and IPTG (0.4 mm) and grown for 16 h at 37 °C. Cells were lysed in a Cell Disruption Systems apparatus at 19 000 psi in Tris**⋅**HCl (50 mm, pH 7.4), imidazole (20 mm) and NaCl (0.5 m). The lysed cells were then centrifuged at 12 000 *g* for 40 min. The supernatant, containing the soluble protein fraction, was transferred onto nickel-loaded chelating sepharose fast flow resin (10 mL) in a 50 mL Falcon tube. The resin was gently agitated for 20 min at 4 °C to allow the His_6_-tagged protein to bind. Centrifugation (4000 *g*, 7 min) yielded the load supernatant, which was removed. The resin was then washed by the addition of washing buffer [Tris**⋅**HCl (50 mm, pH 7.4), imidazole (20 mm), NaCl (0.5 m)]. After thorough mixing the resin was centrifuged at (4000 *g*, 7 min) and the supernatant removed, and this process was repeated a further three times. Elution buffer [20–30 mL, Tris**⋅**HCl (50 mm, pH 7.4), imidazole (0.5 m), NaCl (0.5 m)] was then added to the resin, after a 1 h incubation period (with agitation at 4 °C) this was separated from the resin by centrifugation (4000 *g*, 7 min), yielding the purified protein in the supernatant. The eluted protein was then dialysed against Tris**⋅**HCl buffer (20 mm, pH 7.4). Further purification was achieved by size-exclusion chromatography using an S-200 gel-filtration column (GE Healthcare Life Sciences) in the same buffer before concentration and storage. Protein purity was assessed by SDS-PAGE, and molecular weight was confirmed with ESI-MS. The purified enzymes were usually stored as lyophilised powders before chemical modification.

**Kinetic analyses:** Kinetic parameters of the aldol cleavage reaction were determined at 30 °C using a standard coupled assay^22^, ^38^ with lactate dehydrogenase (LDH) and NADH. The reaction (1 mL final volume) contained varying volumes (2–300 μL) of substrate [Neu5Ac, 100 mm in Tris**⋅**HCl (50 mm, pH 7.4)], LDH (0.5 units), NADH (0.2 mm) and Tris**⋅**HCl (50 mm, pH 7.4). NAL samples, in Tris**⋅**HCl (50 mm, pH 7.4), were added in volumes of 10–200 μL. The decrease in absorbance at 340 nm was recorded on a Uvikon 930 spectrophotometer as the measure of enzyme activity. The rate of substrate cleavage was calculated using a molar extinction coefficient of NADH of 6220 m^−1^ cm^−1^. Kinetic parameters were estimated by fitting the data to the Michaelis–Menten equation.

The pH-activity profiles of the wild-type and K165-γ-thialysine *S. aureus* NAL were obtained at 30 °C using the same assay but with an acetic acid/MES/Tris three-component buffering system covering the pH range 4–9 whilst maintaining a constant ionic strength.[39] Steady-state parameters were measured by fitting the data to the Michealis–Menten equation. The values of *k*_cat_/*K*_m_ against pH were then fitted to a standard bell-shaped curve by using the equation:
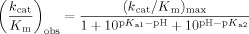


**Mass spectrometry:** Samples were prepared using PD10 desalting columns and then analysed in acetonitrile/1 % aq. formic acid (50:50 *v*/*v*) by nano-electrospray ionisation MS using a quadrupole-ion mobility spectrometry-orthogonal time-of-flight spectrometer (Synapt HDMS, Waters, Manchester, UK). The MS was operated in positive TOF “V” mode using a capillary voltage of 1.2 kV, cone voltage of 50 V, nano-electrospray nitrogen gas pressure of 0.1 bar and backing pressure of 1.78 mbar. The source and desolvation temperatures were set at 80 °C and 150 °C, respectively. Nitrogen was used as buffer gas at a pressure of 8.0×10^−3^ mbar in the trap and transfer regions and 3.6×10^−4^ mbar in the ion mobility cell. Mass calibration was performed by a separate injection of sodium iodide at a concentration of 2 μg μL^−1^ in acetonitrile/water (50:50 *v*/*v*). Data processing was performed using the MassLynx v4.1 suite of software supplied with the mass spectrometer.

**Chemical modification of K165C to yield γ-thialysine:** Lyophilised K165C *S. aureus* NAL (2.5 mg) was dissolved in of prewarmed (37 °C) sodium phosphate buffer, (1.25 mL, 50 mm, pH 8.0) containing urea (6 m). A solution of 2,5-dibromo-1,6-hexadiamide (diBr, synthesised as described;[16] 0.13 mg μL^−1^, 97 μL) in DMF was added to the protein, and was mixed immediately using a vortex mixer for 30 s. The protein/diBr solution was incubated for 1.5 h at 37 °C, with agitation at 200 rpm. An aliquot (70 μL) was then desalted into ammonium acetate buffer (20 mm, pH 7.0), and analysed by ESI-MS to ensure complete conversion of K165C into K165Dha. The conjugate addition of 2-aminoethanethiol onto the K165Dha protein was then carried out on the unfolded K165Dha NAL. A solution of 2-aminoethanethiol (0.1 mg mL^−1^, 40 μL) in Tris**⋅**HCl buffer (1.5 m, pH 8.8) was added to K165Dha NAL (2 mg mL^−1^, 1 mL) and was incubated for 2 h at 37 °C, with agitation at 200 rpm. ESI-MS was used to check for complete conversion of Dha to the γ-thialysine. For determining the pH-activity profile for the thialysine-containing NAL, the above procedure was scaled up so that 50 mg of enzyme was modified.

**Protein refolding:** Refolding of the modified enzyme was carried out in two stages. The modified protein was first dialysed into Tris**⋅**HCl buffer (50 mm, pH 7.4) containing urea (6 m) to remove excess modification reagents, followed by dialysis into the same buffer in the absence of urea to refold the enzyme. Size-exclusion chromatography was performed using an ÄKTA Prime purification system (GE Healthcare Life Sciences) with a Superdex S200 column. Protein (8 mg mL^−1^, 5 mL) was injected onto the column which was run at 2 mL min^−1^.

**Circular dichroism:** CD spectra were obtained at room temperature using a Chirascan CD spectrometer (Applied Photophysics, Leatherhead, UK). All spectra were recorded in a quartz cuvette with path length 1.0 mm. Protein samples were prepared in sodium phosphate buffer (50 mm, pH 8.0) with or without urea (6 m).

**Protein crystallisation:** Crystallisation conditions for the *E. coli* NAL had been previously established[29] and these were used to crystallise the *S. aureus* NAL. Crystals were grown at 18 °C by hanging drop vapour diffusion. A ratio of 2 μL of protein (8 mg mL^−1^) to 2 μL mother liquor was used. The crystallisation conditions were Tris**⋅**HCl (100 mm, pH 7.0–8.5), NaCl (200 mm) and 18–28 % (*w*/*v*) poly(ethylene glycol) (PEG) 3350.

To form the pyruvate complexes of the wild-type and K165-γ-thialysine *S. aureus* NALs crystals were soaked in the mother liquor containing sodium pyruvate (100 mm) and 15 % (*v*/*v*) PEG 400 for 1 min before being sequentially transferred to mother liquor with 5 % increments in PEG 400 concentration. The final soak contained the mother liquor containing sodium pyruvate (100 mm) and 25 % (*v*/*v*) PEG 400. Crystals were then flash-cooled in liquid nitrogen prior to data collection.

**Data collection and refinement:** Diffraction data for all structures were collected from single crystals at the Diamond Light Source macromolecular crystallography beam lines I02 and I04-1. The temperature for data collection was 100 K. Integration and scaling of data was carried out by MOSFLM[40] and SCALA.[41] The structure of the wild-type *S. aureus* enzyme was solved by molecular replacement in Phaser[42] using the structure of *H. influenza* NAL (PDB ID: 1F74)[30] as the search model and subsequent structures were solved by direct Fourier methods. REFMAC5[43] was used for refinement of the data and after each refinement cycle model building was performed in COOT.[44] Coordinates and restraint library files for the lysine residue covalently bound to a pyruvoyl moiety (HET code: KPI) were as previously described.[45] Those for the thialysine side chain (HET code: SLZ) and for the thialysine covalently bound to pyruvate (HET code: KPY) were generated using the PRODRG server and were manually edited. The models were validated using the PDB validation server.

## Notes

As a service to our authors and readers, this journal provides supporting information supplied by the authors. Such materials are peer reviewed and may be re-organized for online delivery, but are not copy-edited or typeset. Technical support issues arising from supporting information (other than missing files) should be addressed to the authors.
