# Supplementary material for: Structural Insights into the Recovery of Aldolase Activity in N-Acetylneuraminic Acid Lyase by Replacement of the Catalytically Active Lysine with γ-Thialysine by Using a Chemical Mutagenesis Strategy
Source: Chembiochem. 2013 Feb 18;14(4):474–81. doi: 10.1002/cbic.201200714 (PMC3792637; doi:10.1002/cbic.201200714)
Supplement: Supplementary file 1 [file cbic0014-0474-SD1.pdf]

CHEMBIOCHEM

Supporting Information

© Copyright Wiley-VCH Verlag GmbH & Co. KGaA, 69451 Weinheim, 2013

Structural Insights into the Recovery of Aldolase Activity in *N*-Acetylneuraminic Acid Lyase by Replacement of the Catalytically Active Lysine with γ-Thialysine by Using a Chemical Mutagenesis Strategy

Nicole Timms,[a, b] Claire L. Windle,[a, b] Anna Polyakova,[a, b] James R. Ault,[a, b] Chi H. Trinh,[a, b] Arwen R. Pearson,[a, b] Adam Nelson,[a, c] and Alan Berry*[a, b]

cbic_201200714_sm_miscellaneous_information.pdf

Table S1. Steady-state kinetic parameters of cysteine mutants of *E. coli* NAL

E. coli enzyme	K_m (Neu5Ac) [mM]	k_{cat} [min^{-1}]
wild-type NAL	2.7 ± 0.2	260 ± 6
C82S	3.8 ± 0.3	260 ± 6
C119S	2.8 ± 0.5	290 ± 7
C238S	3.3 ± 0.4	380 ± 9
C270S	3.5 ± 0.3	300 ± 8
C82S/C119S	4.2 ± 0.3	300 ± 8
C82S/C119S/C238S/C270S	2.5 ± 0.2	40 ± 0.9
C82A/C119A/C238A/C270A	3.2 ± 0.2	290 ± 7
C82S/C119S/C238S/C270S/K165C	3.7 ± 0.3	13 ± 4.2
C82A/C119A/C238A/C270A/K165C	1.5 ± 0.4	3.9 ± 0.3
K165C	5.2 ± 0.3	0.2 ± 0.1

Steady-state kinetic parameters for the cleavage of Neu5Ac by wild-type and cysteine variants of *E. coli* NAL determined using an LDH coupled enzyme assay at pH 7.4.(*21, 38*) Data were fitted to the Michaelis-Menten equation and the fitted values ± standard error of the fit are shown.

Table S2 Primer sequences and conditions for PCR

E. coli C82S forward mutagenic primer<br>5'-TCG CCC ACG TCG GTT CCG TCA GCA CCG C-3'
E. coli C82S reverse mutagenic primer<br>5'-CTT TCA GCT TTG AAG AAC ACT CCG ATC ACT ATC GGG CAA TTA-3'
E. coli C119S forward mutagenic primer<br>5'- CTT TCA GCT TTG AAG AAC ACT CCG ATC ACT ATC GGG CAA TTA -3'
E. coli C119S reverse mutagenic primer<br>5'- GAA AGT CGA AAC TTC TTG TGA GGC TAG TGA TAG CCC GTT AAT -3'
E. coli C238S forward mutagenic primer<br>5'-CGC GCA GAA ACT GCA AAC TGA ATC CAA TAA AGT CAT TGA TTT ACT G -3'
E. coli C238S reverse mutagenic primer<br>5'- CAG TAA ATC AAT GAC TTT ATT GGA TTC AGT TTG CAG TTT CTG CGC G-3'
E. coli C270S forward mutagenic primer<br>5'-GTT TCT GTG CCG CTG TCC CGC AAA CCG TTT GG -3'
E. coli C270S reverse mutagenic primer<br>5'-CCA AAC GGT TTG CGG GAC AGC GGC ACA GAA AC -3'
S. aureus forward cloning primer<br>5'-TCA GAG GAA TTC ATG GAA **CAC CAT CAC CAT CAC CAT** AAC AAA GAT TTA AAA GGT-3'<br>(encoded His_6 tag emboldened; *Eco*RI restriction site underlined)
S. aureus reverse cloning primer<br>5'-GCC GTC ACG TGC CTG CAG CTA TAA ATC GTA TTT TGC AAT-3'<br>(PstI restriction site underlined)
K165C mutagenesis forward primer<br>5'-CAT GAA AAA ATT GTT GGT GTT TGC TAT ACA GCG CCA AAC TTC TTC CTA CTC G-3'
K165C mutagenesis reverse primer<br>5'-CGA GTA GGA AGA AGT TTG GCG CTG TAT AGC AAA CAC CAA CAA TTT TTT CAT G-3'

PCR conditions for amplification of *S. aureus* NAL

The PCR reaction contained 5 μL each of 10 μM forward and reverse primers, 5 μL 0.1ng/μL genomic DNA template, 5 μL 2mM dNTPs, 5 μL of 10x *Pfu* buffer (supplied with the *Pfu* polymerase) and 25 μL of water in a thin walled PCR tube. This was heated for 5 min at 95 ˚C. 0.5 μL of Pfu turbo polymerase (2.5 U/μL) was then added to each reaction, and the following cycling conditions were then used:

95 ˚C for 1 min; 10 repeats of (95 ˚C 1 min, 50 ˚C 1 min, 72 ˚C 2 min) followed by 20 repeats of (95 ˚C 1 min, 60 ˚C 1 min, 72 ˚C 2 min) and finally 72 ˚C for 10 min.

Figure S3 ESI-Mass spectra of modified *S. aureus* NAL

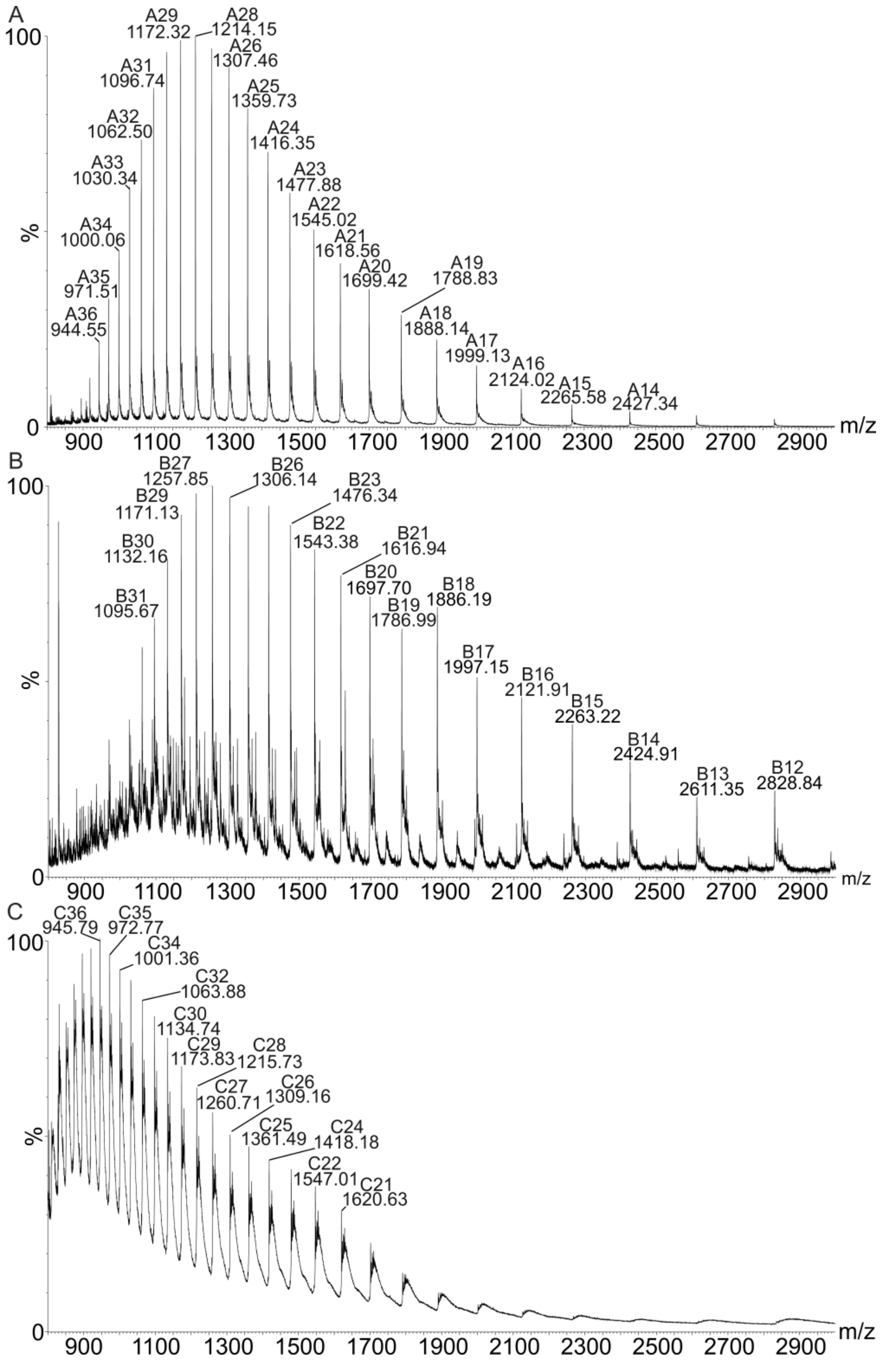

ESI-mass spectra of (A) the K165C variant of *S. aureus* NAL; (B) the K165C variant after 1.5 hrs treatment with 2,5-dibromo-1,6-hexadiamide; and (C) the K165Dha enzyme. These spectra on the m/z scale are deconvoluted onto the mass scale shown in Fig 2.

Figure S4: Tryptic digestion, peptide purification and sequence analysis by ESI-MS

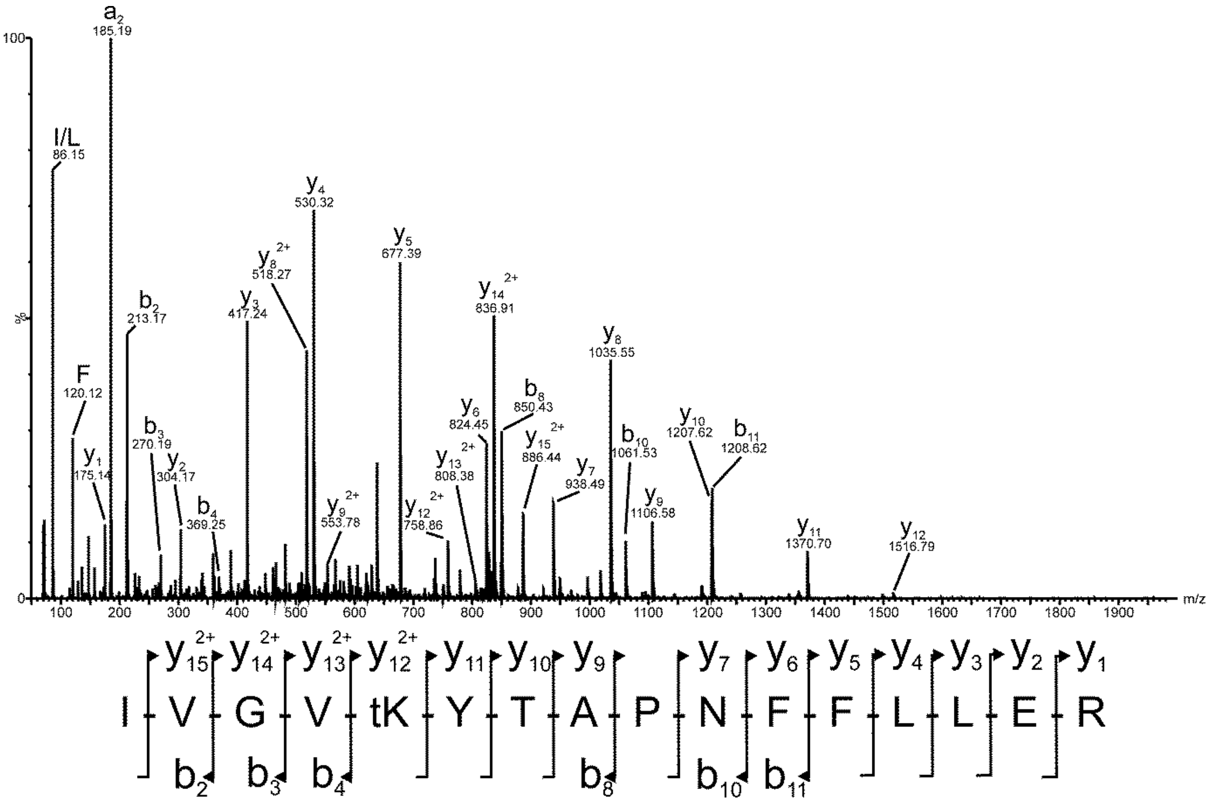

LC-ESI_MS/MS spectrum of m/z 628.9 at tR=26.7 min from the tryptic digest of the chemically modified enzyme showing the structurally diagnostic b- and y-ions demonstrating the presence of γ-thialysine (tK) at position 165.

Gel processing and tryptic digestion.

S. aureus NAL was run on an SDS-PAGE gel and bands were excised and cut into small pieces (~ 1 mm^3), covered with 30 % (v/v) ethanol in a 1.5 mL microcentrifuge tube and heated to 70 °C for 30 min with shaking. The supernatant was removed and replaced with fresh ethanol solution which was again heated to 70 °C for 30 min. This was repeated until all Coomassie stain was removed from the gel. The destain solution was replaced with 50 µL 25 mM ammonium bicarbonate and was shaken for one hour. The wash solution was discarded and the gel slices covered with 25 mM ammonium bicarbonate/50% (v/v) acetonitrile and vortexed for ten mins. The gel slices were then covered with 100% acetonitrile and left for five mins with vortexing before the supernatant was discarded and replaced with a fresh aliquot of acetonitrile. Acetonitrile was removed and the gel pieces were completely dried under vacuum centrifugation for 30 mins. Once dry, the gel slices were cooled on ice. The gel slices were then covered with ice cold trypsin solution (20 ng μL^{-1} in 25 mM ammonium bicarbonate) and left on ice for 30 mins to rehydrate. Excess trypsin solution was removed and the gel slices were covered with a minimal amount of 25 mM ammonium bicarbonate. After briefly vortexing and centrifuging, the gel slices were incubated at 37 °C with shaking for 18 hrs. The resulting digest was vortexed, centrifuged and 50 µL water was added. Following vortexing for 10 mins, the supernatant was recovered and added to an Eppendorf tube containing 5 µL acetonitrile/ water/ formic acid (60/35/5; v/v). 50 µL acetonitrile/ water/ formic acid (60/35/5; v/v) was added to the gel slices and vortexed for an additional 10 mins. The supernatant was pooled with the previous wash and one additional wash of the gel slices was performed. The pool of

three washes was dried by vacuum centrifugation. The peptides were reconstituted in 20 µL acetonitrile/ water/ formic acid (2/97.9/0.1; v/v).

Liquid chromatography-mass spectrometry.

LC-MS/MS analysis of the peptide mixtures was performed on an Ultimate 3000 nano LC system (Dionex, Amsterdam, The Netherlands). 2 µL of each sample in water was loaded onto a C18 guard column and washed with 2% acetonitrile/0.1% formic acid for 5 min at 25 µL min^{-1}. After valve switching, the peptides were then separated on a PepMap C18, 100 µm i.d. x 15 cm analytical column (Dionex, Amsterdam, NL) by gradient elution of 2-60% solvent B in A over 60 min. at 0.3 µL min^{-1}. Solvent A was 0.05% formic acid in 98% water/2% acetonitrile, solvent B was 0.05% formic acid in 20% water/80% acetonitrile.

The column eluant was directly interfaced to a quadrupole-ion mobility- orthogonal time of flight mass spectrometer (Synapt HDMS, Waters UK, Manchester) via a Z-spray nanoflow electrospray source. The MS was operated in positive TOF mode using a capillary voltage of 3.2 kV, cone voltage of 25 V, backing pressure of 2.47 mbar and a trap bias of 4 V. The source temperature was 80°C. Argon was used as the buffer gas at a pressure of 5.0×10^{-4} mbar in the trap and transfer regions. Mass calibration was performed by a separate injection of sodium iodide at a concentration of 2 µg/ µl. GluFib was infused as a lock mass calibrant with a one second lock spray scan taken every 30 s during acquisition. Ten scans were averaged to determine the lock mass correction factor. Data acquisition was using data dependent analysis with a one second MS over *m/z* 350-3000 being followed by three 1 s MS/MS taken of the three most intense ions in the MS spectrum. CE applied was dependent upon charge state and mass of the ion selected. Dynamic exclusion of 60 s was used. Data processing was performed using MassLynx v4.1. Peptide MS/MS data were processed with ProteinLynx Global Server (Waters Ltd, U.K.) and searched against the UniProtKB/SwissProt database (release 2011_12).

Table S5: Structural data

	Wildtype	Wildtype + pyruvate	K165C	γ-thia-lysine	γ-thia-lysine + pyruvate
PDB accession code	4ahp	4ah7	4ahq	4aho	4ama
Diamond beamline station	I02	I02	I04-1	I02	I02
Space group	$P2_12_12_1$	$P2_12_12_1$	$P2_12_12_1$	$P2_12_12_1$	$P2_12_12_1$
a (Å)	82.5	82.3	82.2	82.3	82.9
b (Å)	109.6	109.9	110.0	109.7	110.3
c (Å)	131.3	131.7	133.7	131.4	132.2
Resolution (Å)	46.52-2.1	58.91-2.3	39.27-1.95	29.43-2.00	29.63-2.35
R_{merge} §*	0.075(0.350)	0.117(0.361)	0.067(0.47)	0.064(0.435)	0.11(0.498)
R_{pim} +*	0.044(0.20)	0.065(0.201	0.030(0.225)	0.036(0.248)	0.065(0.289)
Observed reflections	258,615	212,634	499,678	320,723	193,152
Unique reflections	68,405	53,415	86,766	80,641	50,421
Completeness (%)*	97.7(92.8)	99.4(100)	97.8(93.4)	99.6(100)	98.5(99.0)
Multiplicity *	3.8(3.7)	4.0(4.0)	5.8(5.1)	4.0(4.1)	3.8(3.9)
$<I/\sigma>(I/\sigma)$*	10.8(3.4)	9.7(3.7)	14.9(3.2)	14.6 (3.5)	11.4(3.2)
**Refinement**					
R_{factor} (%)	0.1960	0.2050	0.1905	0.1957	0.1894
R_{free} (%) †	0.2476	0.2592	0.2381	0.2353	0.2438
No. of protein atoms	9015	9189	9222	8922	9261
No. of solvent molecules	381	130	868	362	356
No. of ligand atoms	-	20	-	-	20
Average overall *B*-factor ($Å^2$)	25.8	17.9	31.4	33.1	27.7
Average ligand *B*-factor ($Å^2$)	-	13.5	-	-	26.6
Average solvent *B*-factor ($Å^2$)	26.8	14.3	35.8	31.9	22.8
RMS bond lengths (Å) ξ	0.013	0.013	0.015	0.013	0.013
RMS bond angles (°) ξ	1.441	1.617	1.78	1.441	1.586
**Ramachandran analysis, the percentage of residues in the regions of plot (%) ‡**					
Most favoured	98.8	98.1	99.1	98.7	98.3
Outliers	0	0	1■	0	0

* Values given in parentheses correspond to those in the outermost shell of the resolution range.

§ $R_{merge} = \Sigma_{hkl}\Sigma_i|I_i(hkl)-<I(hkl)>|/\Sigma_{hkl}\Sigma_i I_i(hkl)$

+ R_{pim} - precision-indicating (multiplicity-weighted) R_{merge}, relative to all I+ or I-.

$$R_{pim} = \frac{\Sigma_h\left(\sqrt{\frac{1}{n_h-1}}\right)\Sigma_i |I_{hi}-\langle I_h\rangle|}{\Sigma_h \Sigma_i \langle I_h\rangle}$$

† $R = \frac{\sum |F_{obs} - F_{calc}|}{\sum |F_{obs}|}$

R_{free} was calculated in the same way, but for the free set of reflections.
ξ Based on the ideal geometry values of Engh & Huber (1991) *Acta Cryst A*, 47, 392-400
‡ Ramachandran analysis using the program MolProbity (Lovell *et al.*, 2003, *Prot. Struct. Func. And Genet*, 50, 437-450).
▪ The side-chain of Tyr-111 is in close proximity to Leu-142, Thr-143 and Phe-110 from an adjacent chain causing the phi and psi angles of Tyr-111 to lie in an unfavoured region of the Ramachandran plot.

Table S6. Kinetic parameters of wild-type and thialysine-165 modified *S.aureus* NAL at various pH values.

pH	Wild-type NAL			Thialysine-165 containing NAL		
	k_{cat} (min^{-1})	K_m (mM)	k_{cat}/K_m (min^{-1} mM_{-1})	k_{cat} (min^{-1})	K_m (mM)	k_{cat}/K_m (min^{-1} mM_{-1})
5.0	80 ± 3	4.5 ± 0.4	18 ± 2	19 ± 1.6	4.5 ± 1.0	4.2 ± 1.0
6.0	240 ± 9	4.0 ± 0.4	60 ± 6	31 ± 2.2	3.0 ± 0.6	10.0 ± 2.2
6.2	250 ± 6	3.1 ± 0.2	81 ± 5	28 ± 1.0	2.3 ± 0.3	12 ± 1.4
6.4	230 ± 7	2.3 ± 0.2	100 ± 8	28 ± 0.8	1.9 ± 0.2	15 ± 1.5
6.6	250 ± 8	2.7 ± 0.3	93 ± 9	32 ± 0.9	1.4 ± 0.1	23 ± 2.1
6.8	260 ± 6	2.4 ± 0.2	108 ± 7	29 ± 0.8	0.9 ± 0.09	32 ± 3.1
7.0	240 ± 7	2.5 ± 0.2	96 ± 8	29 ± 0.6	1.0 ± 0.07	29 ± 2.0
7.2	250 ± 5	2.4 ± 0.2	104 ± 7	26 ± 1.9	1.0 ± 0.3	26 ± 1.8
7.4	250 ± 5	2.2 ± 0.1	114 ± 6	26 ± 0.9	1.4 ± 0.2	19 ± 2.3
7.6	200 ± 7	1.9 ± 0.2	105 ± 12	25 ± 0.5	1.6 ± 0.1	16 ± 1.0
7.8	230 ± 6	2.3 ± 0.2	100 ± 7	23 ± 0.4	1.4 ± 0.1	16 ± 1.1
8.0	260 ± 13	2.6 ± 0.3	100 ± 10	25 ± 1.8	2.5 ± 0.4	10 ± 1.7
9.0	57 ± 4	1.5 ± 0.3	38 ± 8	8.6 ± 0.8	3.2 ± 0.7	2.7 ± 0.6

Figure S7. pH activity profiles.

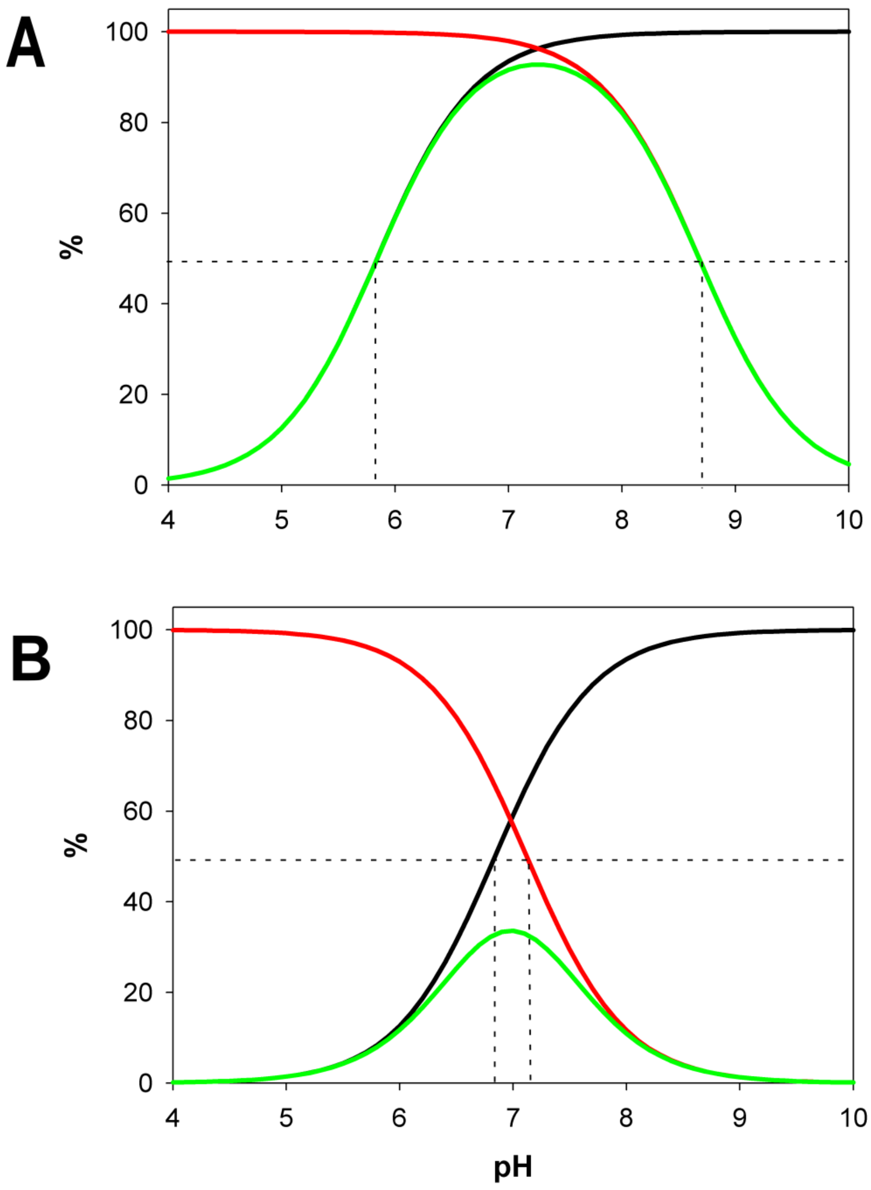

(A) Theoretical pH titration curves showing (red) the % of protonated form of a residue (X) with a p*K*a value of 5.84 and (black) the % of deprotonated form of another residue (Y) with a p*K*a value of 8.68. Assuming that the active form of the enzyme requires the deprotonated form of X and the protonated form of Y, the green line shows the % of the enzyme in the active form. The p*K*a values illustrated are those found for the wild-type *S. aureus* NAL, and shows that almost 100% of the theoretical maximum value of k_{cat}/K_m is experimentally obtained. In contrast when the pKa values of X and Y are 6.84 and 7.12 as illustrated in (B) a maximum of only 33.6% of the enzyme is in the correct ionisation state for activity at the pH optimum.
